# Novel Insights Into the Pathogenesis of Diabetic Cardiomyopathy and Pharmacological Strategies

**DOI:** 10.3389/fcvm.2021.707336

**Published:** 2021-12-23

**Authors:** Felipe Muñoz-Córdova, Carolina Hernández-Fuentes, Camila Lopez-Crisosto, Mayarling F. Troncoso, Ximena Calle, Alejandra Guerrero-Moncayo, Luigi Gabrielli, Mario Chiong, Pablo F. Castro, Sergio Lavandero

**Affiliations:** ^1^Faculty of Chemical and Pharmaceutical Sciences and Faculty of Medicine, Advanced Center for Chronic Diseases (ACCDiS), University of Chile, Santiago, Chile; ^2^Division of Cardiovascular Diseases, Faculty of Medicine, Advanced Center for Chronic Diseases (ACCDiS), Pontifical Catholic University of Chile, Santiago, Chile; ^3^Department of Medical Technology, Faculty of Medicine, University of Chile, Santiago, Chile; ^4^Corporación Centro de Estudios Científicos de las Enfermedades Crónicas (CECEC), University of Chile, Santiago, Chile; ^5^Department of Internal Medicine (Cardiology Division), University of Texas Southwestern Medical Center, Dallas, TX, United States

**Keywords:** diabetes, heart, inflammation, mitochondria, cardiomyopathy, pyroptosis, mitophagy

## Abstract

Diabetic cardiomyopathy (DCM) is a severe complication of diabetes developed mainly in poorly controlled patients. In DCM, several clinical manifestations as well as cellular and molecular mechanisms contribute to its phenotype. The production of reactive oxygen species (ROS), chronic low-grade inflammation, mitochondrial dysfunction, autophagic flux inhibition, altered metabolism, dysfunctional insulin signaling, cardiomyocyte hypertrophy, cardiac fibrosis, and increased myocardial cell death are described as the cardinal features involved in the genesis and development of DCM. However, many of these features can be associated with broader cellular processes such as inflammatory signaling, mitochondrial alterations, and autophagic flux inhibition. In this review, these mechanisms are critically discussed, highlighting the latest evidence and their contribution to the pathogenesis of DCM and their potential as pharmacological targets.

## Introduction

Diabetic cardiomyopathy (DCM) is one of the most severe complications of diabetes. DCM is phenotypically defined as the structural or functional changes of the heart occurring in a diabetic patient independent of other comorbidities such as hypertension, coronary disease, and valvular disease as well as independent of other conventional cardiovascular risk factors. Although the mentioned DCM definition is clear, the specific phenotyping of diagnosed patients is still a matter of discussion mainly due to the potential difference in clinical features among patients. An example of this could be the reported differences in the DCM clinical presentation for the South Asian population in respect to Europeans. Although diastolic impairment expressed as reduced E/A ratio (comparison between the early and late trans mitral flow) was similar, differences in hypertrophy expressed as left ventricular mass and myocardial lipid content were found (1, 2). Another fact pointing in the same direction is that poorly controlled DCM can progress to heart failure (HF). Furthermore, DCM can be featured by either the systolic or diastolic dysfunction, thus generating the so-called HF with reduced ejection fraction or HF with preserved ejection fraction, respectively, causing a substantial detriment to the patient's quality of life (3).

For the clinical presentation, the heart-related data obtained from preclinical animal models of diabetes are also a matter of discussion. One common strategy for generating a type 1 diabetes mellitus (T1DM) model is to induce pancreatic damage using streptozotocin (STZ). At the same time, the most usual approach to develop a T2DM model is to feed animals with a high-fat diet (HFD). HFD and STZ can be used together to mimic T2DM with a stronger hyperglycemic component because of a strong reduction in STZ-induced insulin secretion. Finally, several genetic models of obesity and T2DM, i.e., db/db mice, ob/ob mice, are also used (4).

Considering a variety of models of T1DM and T2DM, the difference in their etiologies generates different functional outcomes in DCM (5). Insulin may play a causal role in the difference. Myocardial insulin signaling in both types of diabetes is quite different as T1DM is characterized by insulin deficiency and T2DM is characterized by insulin resistance. However, in both types of diabetes, a key factor for preventing the progression of DCM to HF is an adequate glycemic control (6). Therefore, despite the different etiologies of both diabetes, several shared molecular alterations are taking place in the myocardium that will be presented as follows.

The aforementioned heterogeneity in the clinical presentation and in the preclinical models of DCM is also projected into the myocardial molecular and cellular physiopathology. Several molecular alterations, such as increased production of reactive oxygen species (ROS), chronic inflammation, fibrosis, mitochondrial dysfunction, autophagic flux inhibition, altered metabolic pathways, altered insulin signaling, cardiomyocyte hypertrophy, and increased myocardial cell death, among others, are described (7). Despite a variety of factors listed earlier, many of them could be subordinated as complementary mechanisms or consequences of broader cellular alterations. However, the approaches that intervene in some of these features, particularly inflammatory signaling, mitochondrial alterations, and autophagic flux, have been described, and hence modifies the DCM phenotype. Therefore, in this review, we focused on the last three mechanisms to outline an updated view of the key mediators in the pathophysiology of DCM. A particular focus is made to propose pharmacological targets to treat DCM. To this aim, we extensively reviewed the data mainly derived from preclinical models, identifying the novel molecular players in the DCM pathophysiology, which have been successfully intervened to achieve the goal of attenuating or improving the DCM phenotype in a structural or functional fashion. The mediators that met the mentioned criteria were considered as pharmacological targets, and the interventions on these pathways were considered as possible pharmacological strategies.

## Inflammation in the Pathogenesis of DCM

Low-grade chronic inflammation is a main contributor to the pathogenesis of DCM. Different DCM-associated stimuli, such as hyperglycemia, hyperinsulinemia, and hyperlipidemia, induce inflammatory signaling pathways, including nuclear factor-kappa b (NF-κB), toll-like receptors (TLRs), inflammasome, and pyroptosis. Also, micro-RNAs (miRNA) and long noncoding RNAs (lncRNA) have been recognized as the regulators of inflammatory signaling in diabetic hearts. Moreover, searching for new targets to modulate the inflammatory response in DCM in cardiomyocytes, fibroblast, endothelium, and vascular smooth muscle cells (VSMCs) has become an interesting research area.

### TLRs in the Pathogenesis of Cardiovascular Effects of Diabetes and Its Implications in DCM

Toll-like receptors have been associated with VSMC damage in cellular models of diabetes (8). Additionally, TLR2 and TLR4 are expressed in endothelial cells under diabetes-associated inflammation (9, 10). A T2DM model obtained by feeding LDL receptor-deficient (LDLR^−/−^) mice with HFD generates large atherosclerotic lesions with increased intimal layer, macrophage, collagen accumulation, and expression of pro-inflammatory cytokines. These alterations are strongly reduced by using the TLR4 antagonist *Rhodobacter sphaeroides* LPS (Rs-LPS) (11). Another inhibitor of TLR4, TAK242, a cell-permeable cyclohexanecarboxylate that directly binds to the Cys747 residue of TLR4 reduces infarct size, edema, hemorrhagic transformation index, and excess hemoglobin. These effects were described in a STZ plus HFD diabetes model, which was subjected to an induced neurovascular injury (12). Also, the viral inhibitory peptide (VIPER), a specific TLR4 blocker, has cardioprotective effects improving diastolic function in a rat model of hypertension induced by angiotensin II (13). Furthermore, in rat VSMC, saturated free fatty acids (FFAs), such as palmitate, activates TLR4 signaling pathway, triggering an inflammatory response, assessed as an increase in monocyte chemoattractant protein 1 (MCP-1) expression (14). Moreover, in human VSMCs, palmitate induces IL-8 expression through the activation of TLR4-NF-κB signaling (15), and also induces apoptosis and oxidative stress through TLR4 pathway (8).

In primary cultured aortic endothelial cells, the depletion of TLR-2 using siRNA prevents the palmitate-induced attenuation of insulin signaling, evaluated as Y612 phosphorylation of insulin receptor substrate 1 (p-IRS1-Y^612^), and blunted insulin-induced endothelial nitric oxide synthase (eNOS) phosphorylation (16). A similar phenomenon is described in wild-type mice fed with HFD for 10 weeks (16). Moreover, in TLR2-KO mice, the insulin-induced mesenteric artery vasorelaxation is unchanged even when fed with HFD (16).

On the other hand, high glucose also induces the TLR2 and TLR4 expression in human macrovascular aortic endothelial cells (HMAECs). This effect is prevented by ROS inhibitors such as N-acetyl cysteine or apocynin (17). Exposure of VSMC to high glucose also triggers TLR4 signaling by increasing the protein level and activity of TLR4, myeloid differentiation factor 88 (MyD88), and NF-κB, augmenting intracellular ROS production. The same findings are found in mesenteric arteries from STZ-induced diabetic rats (18).

In the heart of diabetic mice (STZ and db/db models), Wang et al. showed that advanced glycation end products (AGEs) induce the formation of a complex between myeloid differentiation 2 (MD2) and TLR4. Activated TLR4 triggers a signaling cascade that increases cytokine levels responsible for the development of DCM (19). In neonatal rat cardiomyocytes, oxidized low density lipoproteins (ox-LDL) also induce TLR4 and NF-κB-dependent apoptosis (20). Moreover, global silencing of TLR4, using a systemic administration of TLR4 siRNA, prevents a decline in the systolic function and the ventricular remodeling reported for STZ-treated mice (21). Therefore, TLRs can be considered as a molecule of interest in the pathophysiology of DCM. Although the specific cardiac effects of TLRs are still under investigation, more research is required to clarify the fundamental role of TLRs in the development of DCM. From a pharmacological point of view, it will be interesting to assess whether TLR inhibitors are safe and effective in preventing DCM in several animal models of diabetes.

### Inflammasome Complexes in the Pathogenesis of DCM

Inflammasome complexes are composed of a receptor that is activated by pathogen-associated molecular patterns (PAMPs) or damage-associated molecular patterns (DAMPs), an adaptor protein, and effectors that initiate the inflammatory signaling (22). Nucleotide-binding oligomerization domain, leucine-rich repeat, and pyrin domain-containing protein (NLRP) subfamily are the receptors characterized by a pyrin-containing domain involved in the inflammasome formation. After the binding of PAMPs or DAMPs to NLRPs, the pyrin domain interacts with the pyrin domain of the apoptosis-associated speck-like protein (ASC), which activates bound pro-caspase-1 to caspase-1, leading to the secretion of IL-1β and IL-18, or pyroptosis activation (22).

NLRP3 inflammasome is activated in HFD + STZ diabetic rats (23), where there is an increase in NLRP3, ASC, pro-caspase-1, caspase-1, pro-IL-1β, and IL-1β protein levels (23). The expression of sirtuin 3 (SIRT3), a protein member of class III histone deacetylases dependent on nicotinamide adenine dinucleotide (NAD^+^), is decreased in the myocardial samples of STZ-induced diabetic mice, affecting cell energy metabolism and activating NLRP3 inflammasome. The SIRT3 KO animals display ventricular diastolic and systolic dysfunction, and necroptosis. Interestingly, cardiac dysfunction is prevented by inhibiting the NLRP3 inflammasome activation (24). The inhibition of miR-223, a miRNA expressed in diabetes, downregulates the markers of inflammation (NLRP3), fibrosis (collagens I and III), and apoptosis (caspase-3 and Bax), attenuating the cardiac effects as well in the systolic function and ventricular remodeling of STZ-induced diabetes (24). The same effect is obtained by metformin. In hearts from STZ-treated mice, metformin inhibits the increases in NLRP3, caspase-1, and IL-1β in an AMP-activated protein kinase/mammalian target of rapamycin (AMPK/mTOR-) dependent manner (25). Melatonin reduces cardiac fibrosis and improves cardiac systolic function in diabetic mice *via* inhibiting lncR-MALAT1/miR-141-mediated NLRP3 inflammasome activation (26). Another widely used treatment for T2DM is the combination of sodium-glucose cotransporter-2 inhibitors (SGLT2i) with dipeptidyl peptidase-4 (DPP-4) inhibitors (27). The treatment with dapagliflozin (an SGLT2i) and saxagliptin (a DPP-4 inhibitor) has an additive effect on the cardio protection of BTBR *ob/ob* diabetic mice by decreasing NLRP3 protein levels (28). In BTBR *ob/ob* mice, the combination of dapagliflozin with ticagrelor (P2Y12 receptor antagonist) also reduces cardiac hypertrophy, apoptosis, inflammation, fibrosis, and NLRP3 inflammasome markers associated with DCM (29).

Gypenosides (Gps), the main active compound in the herbaceous plant *Gynostemma pentaphylla Makino*, decrease the NLRP3 inflammasome activation in hearts from STZ + HFD-treated rats and exerts anti-inflammatory properties improving myocardial histological changes associated to DCM although there is a lack of cardiac functional data associated with the treatment (30). Visceral adipose tissue-derived serine protease inhibitor (vaspin) decreases NLRP3 levels in an autophagy-dependent mechanism in STZ-induced diabetes model, associated with the improvement of a systolic function (31). Finally, rosuvastatin, a 3-hydroxy-3-methylglutaryl coenzyme A reductase inhibitor, can alleviate the consequences of diabetes in rat hearts through the downregulation of NLRP3 (32). Other modulators of NLRP3 expression in STZ-treated diabetic rats are Spleen tyrosine kinase (SYK) and c-Jun N-terminal kinase (JNK). The treatment with the JNK inhibitor (SP) and the SYK inhibitor (BAY61-3606) inhibits NLRP3 activation in cellular models (33).

Another subfamily of the inflammasome complex is absent in melanoma 2 (AIM2), a protein that participates in inflammasome formation in the presence of cytoplasmic DNA (34). AIM2 is increased in the heart from STZ rats, and AIM2 knockdown attenuates cardiac hypertrophy accompanied by improvements in the diastolic and systolic function (35).

Only a few studies have addressed the activation of NLRP3 inflammasome in human heart samples. Fender et al. showed that right atrial appendages from patients with T2DM display higher levels of PAR4 than nondiabetic atrial tissue, along with an increased abundance of cleaved caspase-1, IL-1β and the plasma membrane pore-forming protein N-terminal gasdermin D, a protein required for IL-1β secretion (36). Immunohistochemistry evaluation of heart tissue samples obtained from the failing human hearts at the time of transplantation shows an increase in caspase-1 expression in human diabetic hearts as compared to nondiabetic heart tissue sections. The increase in caspase-1 expression is associated with a higher infiltration of inflammatory cells into the myocardium of diabetic heart tissues (37). These results suggest that in human hearts, as in animal models, NLRP3 inflammasome is activated in DCM.

**Figure 1:**
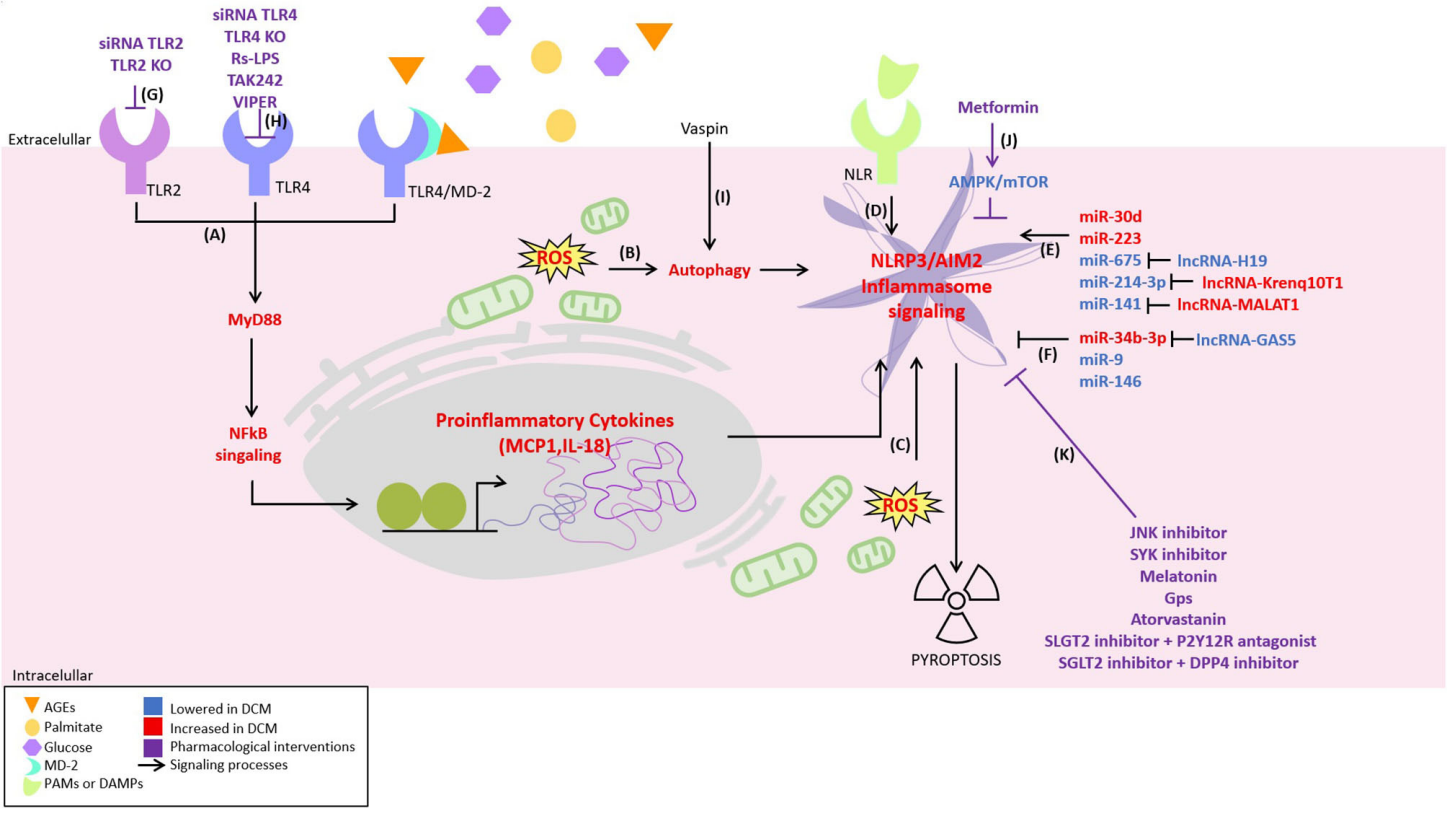
Novel inflammatory signaling pathways implicated in diabetic cardiomyopathy (DCM) pathogenesis and its possible pharmacological interventions. Inflammatory signaling has been proposed to be responsible for the development of DCM. It is activated by different pathways, including: **(A)** nuclear factor-kappa b (NF-κB) signaling activated by toll-like receptor 2 (TLR2), TLR4, and myeloid differentiation 2 (MD2)/TLR4 complex, **(B)** oxidative stress-induced autophagy, **(C)** oxidative stress, **(D)** activation of the NLR by pathogen-associated molecular patterns (PAMPs) or damage-associated molecular patterns (DAMPs). Moreover, some non-coding RNAs are described to **(E)** positively or **(F)** negatively regulate the inflammasome NLPR3. On the other hand, possible therapeutical strategies attenuating inflammatory signaling are illustrated as follows: **(G)** TLR2 depletion using siRNA TLR2 or TLR2-KO. **(H)** TLR4 depletion using siRNA TLR4 or TLR4-KO, TLR4 antagonist *Rhodobacter sphaeroides* LPS (Rs-LPS) and TLR4 blocker TAK242 and viral inhibitory peptide (VIPER), **(I)** decreasing NLRP3 by vaspin in an autophagy-dependent mechanism, **(J)** the inhibition of NLRP3 by metformin in an AMPK/mTOR-dependent manner, and **(K)** c-Jun N-terminal kinase (JNK) and spleen tyrosine kinase (SYK) inhibitor, melatonin, gypenosides (Gps), atorvastatin, SLGT2 inhibitor + P2Y12R antagonist, and SGLT2 inhibitor + DPP4 inhibitor.

Despite a great variety of molecules used in several diabetes animal models to reduce NLRP3/AIM2 inflammasome activation (Figure 1), and hence, to prevent cardiac injury, clinical trials are still required to study the possible benefit of NRLP3 targeted therapies in humans.

### Pyroptotic Cell Death in the Pathogenesis of DCM

Many of the studies have linked the different types of cell death to the development of DCM. Within the inflammatory context, the different types of programmed necrosis predominate, including pyroptosis (26, 38, 39). Several pharmacological interventions have been tested in DCM models (40). Exendin-4, an analog of the incretin GLP-1 used to treat diabetes, prevents the systolic dysfunction in HFD-fed mice, also reduces the markers of pyroptosis, such as caspase-1 activation and IL-1β and IL-18 release, in mice cardiomyocytes exposed to high glucose (33). Chemerin is an adipokine that is upregulated in obesity and diabetes and has been associated with cardiovascular disorders (41, 42). Chemerin and its receptor CMKLR1 are increased in the cardiac tissue of HFD + STZ rats, along with the markers of pyroptosis, such as NLRP3, cleaved caspase-1, and IL-1β (43). CMKLR1 knockdown reduces pyroptosis markers in the heart tissue of diabetic rats and reduces cardiac fibrosis and hypertrophy associated with an improvement in cardiac systolic and diastolic function (43).

In diabetic hearts, noncoding RNAs are described as the regulators of pyroptosis. Using neonatal rat cardiomyocytes treated with high glucose and hearts from STZ + HFD mice, Li et al. demonstrated the presence of pyroptosis, which was mediated by the upregulation of micro-RNA-30d (miR-30d) (44). miR-30d directly represses the expression of FoxO3, which in turn mediates the expression of an apoptosis repressor with caspase recruitment domain (ARC), an endogenous inhibitor of caspase activation. The consequent decrease in ARC levels in these DCM models activates caspase-1 and the pyroptotic pathway (44). miR-214-3p, which targets caspase-1, is also downregulated in diabetic mice and in human cardiomyocytes exposed to a high-glucose (HG) medium, leading to increased levels of caspase-1 and the activation of pyroptosis (45–47). A minimum of two noncoding RNAs endogenously regulate this microRNA, lncRNA Kcnq1ot1 (46, 47), and caspase-1-associated circular RNA (CACR) (45). These two noncoding RNAs are competitors for miRNA binding, generating a miRNA sponge effect. Kcnq1ot1 is upregulated in the serum of diabetic patients. Silencing this lncRNA reduces pyroptosis both *in vitro* and *in vivo* and ameliorates the systolic dysfunction in diabetic mice (46, 47). Similarly, CACR is also upregulated in HG-treated cardiomyocytes and serum of diabetic patients. Knockdown of CACR in cardiomyocytes reduces both caspase-1 activation and pyroptosis (45). Another example is miR-34b-3p, which is markedly upregulated in a DCM mice model. miR-34b-3p targets the aryl hydrocarbon receptor (AHR), a negative regulator of NLRP3 inflammasome and pyroptosis (48). miR-34b-3p also has an endogenous regulator, the lncRNA GAS5, whose expression is strongly diminished in the hearts of diabetic mice (48). Furthermore, the overexpression of GAS5 reduces pyroptosis markers in the heart tissue and improves a systolic function (48). miR-9 also regulates pyroptosis in the diabetic heart (37). This miRNA is markedly downregulated in human diabetic hearts and HG exposed human cardiomyocytes, leading to the increase of its target, the ELAV-like protein 1 (ELAVL1) (37). ELAVL1 is an RNA-binding protein that stabilizes mRNAs of inflammatory genes, including NLRP3 and caspase-1, causing increased IL-1β production in the cardiac tissue (37). Accordingly, human cardiomyocytes transfected with miR-9 mimics reduced ELAVL1, caspase-1, and IL-1β expression (37).

In total, these studies show that caspase-1-induced pyroptosis in the heart may be an essential mediator of DCM pathogenesis (Figure 1) and could be regulated by noncoding RNAs, offering new therapeutic targets for treating this disease.

### MiRNAs as Regulators of DCM and Its Inflammatory Features

In addition to the role of the abovementioned miRNAs in cardiac pyroptosis, other miRNAs have been described to regulate the different aspects related to inflammation in DCM (49). miR-675, along with its precursor lncRNA H19, is downregulated in STZ rats and neonatal rat cardiomyocytes exposed to high glucose (50). miR-675 targets the mitochondrial protein VDAC, which is increased in the diabetic model, leading to apoptosis of cardiomyocytes. The overexpression of H19 normalizes VDAC levels to reduce apoptosis, inflammation, and oxidative stress of cardiac tissues and improves cardiac systolic and diastolic function (50). Cardiac levels of miR-146a are also decreased in STZ-treated mice, specifically in endothelial cells, but not in cardiomyocytes (51). Endothelial-specific overexpression of miR-146a in diabetic mice reduces inflammatory markers such as IL-6, IL-1β, TNF-α, and p65-NF-κB, reducing cardiac fibrosis and diastolic dysfunction (51). Furthermore, a downregulation of miR-181a-5p, due to its regulator KCNQ1OT1, in both HG-cultured cardiomyocytes and STZ-treated mice is observed. However, its overexpression decreases inflammatory cytokine levels, myocardial apoptosis, and fibrosis *in vivo*, thus improving the DCM phenotype.

Cardiac fibrosis is also a characteristic of DCM, where cardiac fibroblasts show an activated phenotype with a greater production of extracellular matrix, a process regulated by the TGF-β1 and Smad2/3 signaling pathway. Neonatal cardiac fibroblasts exposed to a HG medium show increased TGF-β1 signaling and the production of collagens I and III with a parallel increase in miR-150-5p levels (52). miR-150-5p reduces Smad7 expression, a protein that binds to a TGF-β1 receptor and prevents the activation of a Smad2/3 pathway. Silencing miR-150-5p in cardiac fibroblast restored Smad7 levels and prevented the increase in pro-fibrotic and pro-inflammatory markers *in vitro* (52). miR-223 has also been associated with cardiac inflammation and fibrosis in STZ-induced diabetic rats (24). The silencing of miR-223 reduces fibrosis, NLRP3 expression, and apoptosis and ameliorates cardiac function in diabetic rats (24).

Menopause has been shown to aggravate DCM in women. To simulate postmenopausal estrogen deficiency in older diabetic women, Jia et al. treated ovariectomized (OVX) mice with STZ. A worsening of cardiac function compared to mice subjected to STZ alone is found (53). This cardiac dysfunction is associated with an imbalance between pro-inflammatory type 1 macrophages (M1) and anti-inflammatory type 2 macrophages (M2) infiltrated in the heart tissue. miR-155, which has been previously linked to pro-inflammatory imbalance during viral myocarditis (54), is markedly upregulated in OVX/STZ mice (53). Interestingly, the delivery of gold nanoparticles containing antagomiR-155, which are preferentially phagocytosed by macrophages, counteracts M1/M2 imbalance and restores cardiac function, reducing cardiac hypertrophy, fibrosis, and apoptosis of cardiac cells (53).

A 5-year longitudinal study on patients with T2DM, compared with nondiabetic age-matched controls, showed an increase in cardiac hypertrophy associated with a progressive impairment in cardiac strain that was paralleled by the upregulation of miR122-5p (55). The miRNA regulates macrophage polarization (56) and mediates inflammation in a model of acute lung injury induced by lipopolysaccharide (57). Interestingly, miR-208a, described originally to be increased in a mouse model of cardiac hypertrophy (58), also regulates the synthesis of IL-10 in human macrophages (59). The miRNA is increased in atrial samples derived from diabetic patients (60), suggesting a possible cross-regulation between cardiac hypertrophy and inflammation in DCM hearts. Moreover, the use of an anti-miR-208a in Dahl hypertensive rats prevents cardiac remodeling while improving cardiac function (61).

In total, these studies show that various miRNAs regulate different aspects of inflammation in DCM (Figure 1), through the regulation of numerous target genes. In the near future, understanding how these miRNAs act on the pathogenesis of DCM will offer new therapeutic targets for the treatment of this disease.

## Mitochondrial Alterations in the Pathogenesis of DCM

Mitochondria have been a top research topic for decades. Dysfunctional mitochondria are involved in the pathogenesis of multiple chronic diseases from cancer to neurodegenerative disorders, and DCM is not an exception (62). Alterations in mitochondrial substrate utilization and mitochondrial dynamics have been described as pathological features in the diabetic heart (63). Several mitochondrial approaches to revert, prevent, or attenuate the DCM phenotype have been described. In the following sections, updated evidence about interventions targeting mitochondrial processes and their relevance in the DCM pathogenesis will be described.

### Mitochondrial Substrate Utilization as a Pathological Feature in DCM

A healthy heart uses fatty acids as the main source of adenosine triphosphate (ATP) (70%). However, cardiomyocytes can also obtain acetyl-CoA from glucose, ketone bodies, lactate, and amino acids (64). Despite the increased availability of glucose due to the DCM-associated hyperglycemia, a decrease or loss in the capacity to oxidize glucose with a subsequent increase of fatty acid utilization is observed. Fatty acid oxidation uses 12% more oxygen than glucose oxidation (64). Left ventricular myocardium from patients with T2DM and nondiabetic patients undergoing coronary artery bypass graft surgery was obtained using subepicardial needle biopsy. The myocardium samples from patients with T2DM have a decreased mitochondrial respiration fueled by palmitoyl-carnitine. Diabetic myocardium also has a diminished activity of hydroxyacyl-CoA dehydrogenase and accumulates more lipid droplets (65). These results suggest that diabetic hearts exhibit a decreased mitochondrial capacity for β-oxidation with increased accumulation of intracellular lipids. This change in substrate utilization is believed to contribute to cardiac hypertrophy, along with ventricular dysfunction, both cardinal characteristics of DCM.

Recently, to promote glucose utilization in a model of diabetes induced by low-dose STZ, Wende et al. developed a mouse model with an inducible cardio-specific expression of GLUT4 (66). Surprisingly, despite the display of increased glucose uptake and utilization using the transgenic model, no improvement in the DCM phenotype is observed. Moreover, GLUT4 induction increases the diastolic dysfunction associated with the decrease of mitochondrial respiratory complex activity by O-GlcNAcylation (OGA) and reduces the transcript levels of electron transfer chain subunits (66). In STZ + HFD diabetes mice models, hyperglycemia is also associated with reduced cardiac expression of β-hydroxybutyrate-dehydrogenase (BDH1) and succinyl-CoA:3-oxoacid CoA transferase (OXCT1) due to OGA. This phenotype is recapitulated by a dominant-negative transgenic mouse for OGA enzyme responsible for OGA removal (67). Reliable and relevant data about OGA and its implications in the diabetic heart suggest that OGA could be targeted to design a protective intervention to prevent DCM. In this context, substantial attenuation of the structural and functional cardiac changes induced by diabetes is obtained by OGA overexpression (68) although its association with increased ketone utilization remains unclear. On the other hand, the overexpression of BDH1 increases ketone utilization by 1.7 folds and displays protective actions on cardiac structural and functional injury induced by transverse aortic constriction (TAC) surgery, suggesting that increasing ketone body uptake could be beneficial beyond DCM interventions (69). However, the link between ketone metabolism and DCM remains to be fully explored (68).

The CANVAS trial, which analyzed the efficacy and safety of canagliflozin (an SGLT2i) in 10,142 patients with T2DM and high cardiovascular risk, reported that SGLT2i significantly reduced cardiovascular risk and decreased hospitalization due to HF (70). Ferrannini et al. (71) analyzed the EMPA-REG OUTCOME clinical study (72) involving patients with T2DM and cardiovascular risk and found that empagliflozin modulated myocardial energy metabolism increasing the ketone body utilization. These ketone bodies are oxidized in preference to fatty acids, improving the efficiency of mitochondrial metabolism and oxygen consumption (71). These researchers propose SGLT2i as cardioprotective agents with hypoglycemic effects, which are beneficial for DCM (71). Furthermore, Li and Zhou summarize the beneficial impact of SGLT2i on DCM in preclinical studies, highlighting its metabolic, anti-fibrosis, and antioxidative functions (73).

Calpains are upregulated in the diabetic heart and directly affect the protein level and activity of ATP synthase (ATP5A1), decreasing mitochondrial ATP synthesis (74). Calpain inhibition increases the activity of ATP5A1, prevents the mitochondrial ROS, reduces cardiac hypertrophy markers, and improves the myocardial function determined by the increased fractional shortening and recovery of E/A ratio (74). Similarly, in STZ-induced diabetes model, the overexpression of calpastatin (endogenous calpain inhibitor) and the knockout *capn4*, a subunit required for calpain activity, prevents cardiomyocyte apoptosis and ventricular remodeling (75, 76).

In summary, more research is still required to perform pharmacological modulation of mitochondrial metabolism to slow down the progress and development of DCM. Furthermore, based on the recent evidence of ketone glucose utilization by cardiac cells, it remains to fully elucidate whether the metabolic changes are protective adaptations or whether those are the main problems in the pathogenesis of DCM.

### Mitochondrial Oxidative Stress in DCM

Augmented ROS production in the diabetic heart is one of the leading factors in the development of DCM. Therefore, the strategies for reducing ROS or increasing antioxidant mechanisms to improve or maintain myocardial function in diabetes are of significant interest (77). There are several sources of cytosolic ROS, including NADPH oxidases (NOX), xanthine oxidases, and uncoupled nitric oxide synthases (NOS), most of which are involved in the development of DCM and have been reviewed previously (78–81). However, mitochondria are the primary sources of cellular ROS, specifically from complexes I and III of the electron transport chain, where the superoxide radical is generated by the transfer of electrons to molecular oxygen (62). Mitochondrial oxidative stress is triggered when the generation of superoxide increases and/or the defense capacity of the antioxidant system decreases (82, 83). Mitochondrial ROS increase in both STZ-induced T1DM and db/db T2DM models, indicating oxidative stress, which is associated with mitochondrial dysfunction, together with HF and DCM (84–86).

Several mechanisms that trigger mitochondrial oxidative stress associated with DCM have been described so far (82). TOM70 is an outer mitochondrial membrane (OMM) translocase that is downregulated in diabetic hearts. Knockdown of TOM70 generates an exacerbated DCM phenotype in db/db mice and increases cytosolic and mitochondrial ROS *in vitro* in HG/high-fat-treated neonatal mouse cardiomyocytes. Conversely, lentiviral overexpression of TOM70 prevents hypertrophy, fibrosis, and ventricular dysfunction (87). TOM70 participates in the import of several mitochondrial proteins with potential antioxidant effects; however, the mechanism by which TOM70 reduces ROS remains to be fully elucidated.

As mentioned earlier, the diabetic heart exhibits an increase in the oxidation of fatty acids, which leads to mitochondria overload, with the consequent overproduction of ROS (82). Forkhead box protein O 1 (FOXO1) is a transcription factor that participates in the expression of lipid uptake and lipid metabolism-related genes, and it is increased in the heart of genetic (db/db) and HFD-induced diabetes models (88). In STZ-treated mice, FOXO1 indirectly increases NADPH oxidases 4 (NOX4) expression, increasing cytosolic ROS, and increases peroxisome proliferator-activated receptor alpha (PPARα), promoting fatty acid uptake and mitochondrial overload, with a consequent increase in mitochondrial ROS production (89). Accordingly, one study has shown that micro-RNA 30c (miR-30c) is an interesting new therapeutic target for DCM as its overexpression reduces the levels of PGC-1β, which is an important coactivator of PPARα, thus reducing lipid accumulation and ROS production in the heart of db/db mice (90).

**Figure 2:**
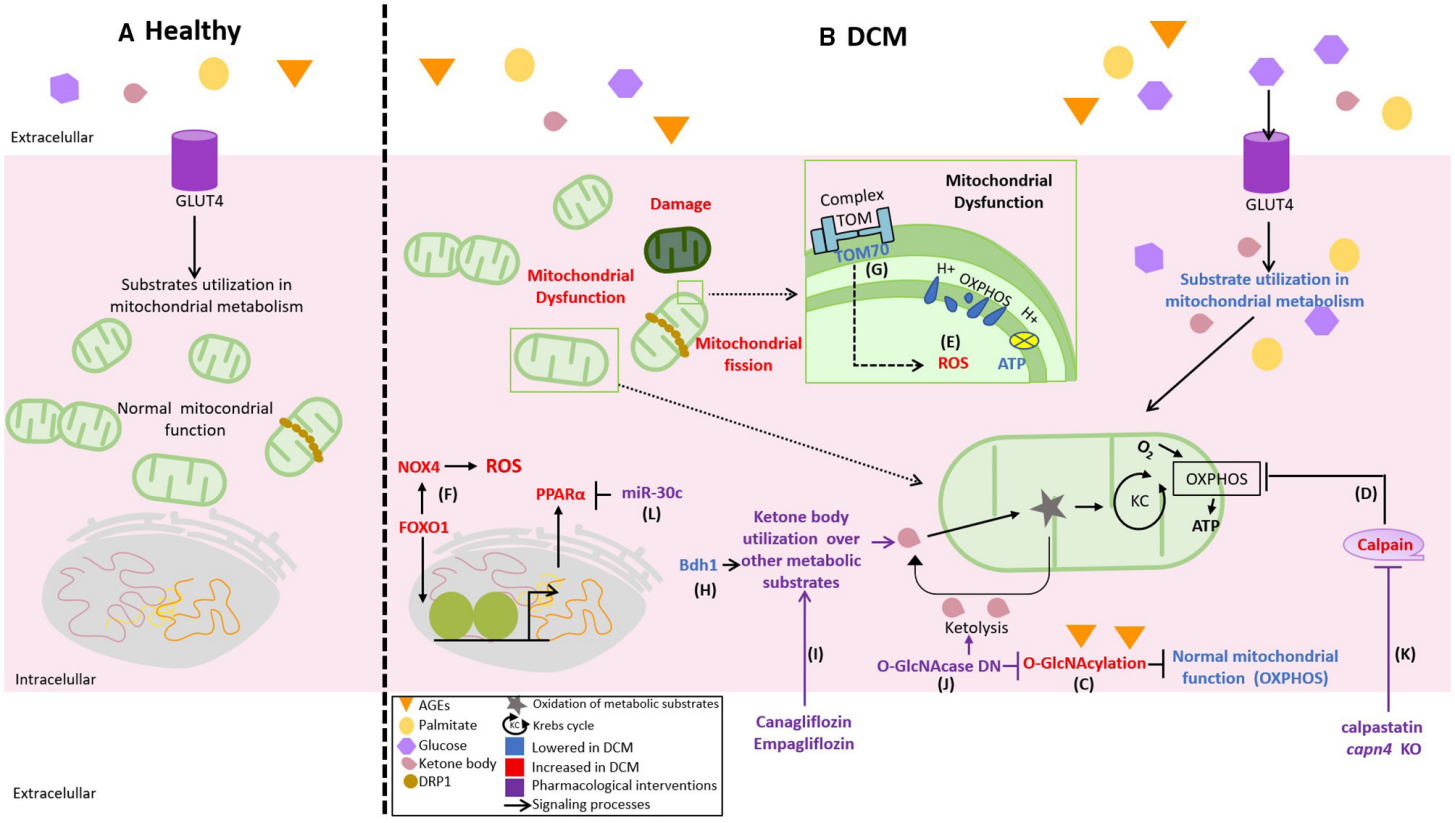
Novel differences between healthy and DCM mitochondrial function and its pharmacological targets. **(A)** Normally, the mitochondria used glucose, lipids, and ketones as the fuel to obtain adenosine triphosphate (ATP) in the oxidative phosphorylation (OXPHOS) according to the necessities of the cell, **(B)** but in DCM, there is an excess of available fuels, increasing glucose, palmitate, and advanced glycation end products (AGEs) that affect the flexibility in substrate use by the mitochondria triggering mitochondrial dysfunction. This process is evidenced by a lower mitochondrial membrane potential, decreased production of ATP by OXPHOS, and increased reactive oxygen species (ROS). The mechanisms of mitochondrial deterioration in DCM are illustrated as follows: **(C)** the decrease in OXPHOS activity by O-GlcNAcylation (OGA), **(D)** the upregulation of calpains in the diabetic heart, **(E)** the abnormal production of mitochondrial ROS, **(F)** the production of cytosolic ROS by an increase in the expression of NADPH oxidases 4 (NOX4) indirectly regulated by FOXO1, and **(G)** the downregulation of TOM70 in the diabetic heart. Thus, therapeutic strategies aiming to improve a mitochondrial function are represented as follows: **(H,I)** the overexpression of β-hydroxybutyrate-dehydrogenase (BDH1) and Canagliflozin or Empagliflozin to increase the use of ketones, **(J)** dominant-negative for OGA (O-GlcNAcase DN), **(K)** inhibition of calpain activity by calpastatin (endogenous calpain inhibitor) or *capn4* knockout, and **(L)** micro-RNA 30c (miR-30c) reduces the proliferator-activated receptor alpha (PPARα) coactivator PGC-1β.

Thus, the studies concerning mitochondrial oxidative stress and DCM seem to involve the antioxidant defense systems and the modulation of upstream proteins involved in superoxide formation by the electron transport chain (Figure 2). However, the underlying mechanisms are still being investigated, but they are a growing source for new therapeutic targets for DCM.

### Mitochondrial Dynamics in the Pathogenesis of DCM

Mitochondria are highly dynamic organelles as they perform coordinated cycles of fission (mitochondrial fragmentation) and fusion (mitochondrial elongation) to regulate shape, size, subcellular distribution, and the number of mitochondria per cell (91). These processes, termed as mitochondrial dynamics, allow rapid and transitory morphological changes that regulate mitochondrial function and cellular processes related to apoptosis, metabolism, mitochondrial quality control, cell cycle, etc. (91). Mitochondrial fusion and fission are controlled by specific machinery. During mitochondrial fusion, mitofusins 1 and 2 (MFN1 and MFN2) and optic atrophic protein 1 (OPA1) maintain the fusion of the OMM and the IMM, respectively (91, 92). Mitochondrial fission depends on the recruitment of the Dynamin-related protein 1 (Drp1) GTPase from the cytosol of the GTPase Drp1 to the OMM by different surface receptor proteins. This binding allows the oligomerization of Drp1 and the constriction of mitochondria until the complete division into two-daughter mitochondria (92). The processes of mitochondrial fusion and fission need to be balanced to maintain a healthy mitochondrial population, which is essential for a normal heart function. Therefore, alterations in mitochondrial dynamics are considered to be one of the crucial mechanisms related to the development of DCM (93, 94).

Diabetic cardiomyopathy has been related to a largely fragmented mitochondrial phenotype. Westermeier et al. reviewed different diabetes models to address a direct relationship between mitochondrial fission/fusion and insulin resistance (63). Hyperglycemia induces mitochondrial fragmentation, increases ROS, and activates apoptotic pathways in neonatal rat ventricular myocytes and H9c2 cells. Hyperglycemia-dependent effects are prevented by a Drp1 dominant-negative, DRPK38A, demonstrating the involvement of mitochondrial fission (95, 96).

Recently, it was described in db/db mice models that the proportion of long and short isoforms of OPA1 (L-OPA1/S-OPA1) is decreased (97). The balance of long and short isoforms is necessary for the fusion of IMM. Therefore, an excess of S-OPA1 or a reduction in L-OPA1 prevents mitochondrial fusion. Moreover, Drp1 and Fis1, a Drp1 receptor protein, are also increased in hearts from db/db mice, which agrees with the observed mitochondrial fragmentation phenotype. Furthermore, as mitochondrial ATP production and oxygen consumption are decreased and ROS production is augmented, these mitochondria are also dysfunctional (97). These changes were prevented by feeding the mice with a ketogenic diet, accompanied by an increase in L-OPA1/S-OPA1 ratio, a drop in Fis-1, and no changes in Drp1 (97). Indeed, the restoration of OPA1 function also improves the systolic function in STZ diabetic rats (98). Therefore, the imbalance in mitochondrial dynamics triggered by an alteration in OPA1 contributes to the development of DCM.

Mitofusin 2 has also been shown to be a potential therapeutic strategy for DCM. In neonatal rat cardiomyocytes, PPARα positively regulates the expression of MFN2 by directly binding to its promoter. The expression of PPARα is decreased in cardiomyocytes cultured in high glucose/high lipid (OXO1) media producing a decrease in MFN2 levels and consequently inducing mitochondrial fission leading to mitochondrial dysfunction (99). *In vivo*, the restoration of MFN2 improved the systolic function and attenuated the diastolic changes associated with DCM (99).

In right atrial myocardium samples obtained from patients with T2DM, MFN1 levels are decreased and myofiber contractibility and oxygen consumption are reduced compared with obese individuals (100). However, this result must be taken cautiously because neither patients with obesity nor T2DM were affected by cardiomyopathy (100). Nevertheless, this result suggests that in the progression of obesity to diabetes, an alteration in mitochondria dynamics and function has taken place.

Therefore, regulating mitochondrial dynamics from the point of view of mitochondrial morphology seems to be an interesting therapeutic target in DCM (Figure 2). Restoring or preventing the downregulation of proteins involved in mitochondrial fusion could normalize the balance between mitochondrial fusion and fission, which is directly related to the improvement in mitochondrial activity and, consequently, in cardiac function.

## Autophagy Alterations in DCM

Autophagy is a cellular catabolic process responsible for recycling macromolecules and futile organelles as part of cellular homeostasis. This process is tightly regulated by autophagy-related genes (ATGs) (101). Three types of autophagy are described. Micro-autophagy occurs when proteins are degraded by direct entry into the lysosome. Chaperone-mediated autophagy, a process where proteins with the recognition sequence KFERQ binds to the chaperone 70 kDa heat shock protein (Hsp70) responsible for transporting these proteins to the lysosome in a lysosome-associated membrane protein 2- (LAMP2-) dependent fashion. Finally, macro-autophagy, commonly referred to as autophagy, implicates the formation of a phagophore, which is a nascent two-layered vesicle that encapsulates cytosolic components; the formed phagophore requires to be maturated and finally fused with the lysosome (101–103).

Several pieces of evidence suggest that autophagy dysregulation is involved in the pathogenesis of DCM (102). Most of the data show that autophagic flux is decreased or blunted in the heart of T2DM models and, conversely, increased in some models of T1DM (101, 104). Indeed, regarding T1DM, there is a recent report showing that FoxO1 mediates over-reactive autophagy, which has a causal role in the pathogenesis of DCM in STZ-treated mice. In these animals, the administration of angiotensin IV suppresses the increased autophagy and attenuates the pathological ventricular phenotype (105). It has been proposed that in T2DM, autophagy is a critical factor in the pathogenesis of DCM, while in T1DM, autophagy is a mechanism to limit the glucotoxicity damage in the heart (104). This dichotomy about the role of autophagy in diabetes is still a matter of discussion.

### Decreased Autophagic Flux as a Feature of DCM

As described earlier, in STZ treatment-induced T1DM models, the autophagic flux in the heart is induced (104). However, chronic T1DM (6 months) also causes autophagic flux inhibition (106). The same authors reported that cardiomyocyte-specific ATG5 knockout imitates the autophagic inhibition of the long-standing STZ approach and accelerates the progression of the heart dysfunction through a nuclear factor-erythroid factor 2-related factor 2- (Nrf2-) dependent mechanism. Nrf2 knockout rescues the phenotype induced by the deletion of ATG5 gene, concluding that Nrf2 could exert deleterious aberrant signaling once autophagy is inhibited (106). The final results are very interesting as Nrf2 has been proposed to be a therapeutic strategy for DCM due to a plethora of antioxidant and anti-inflammatory genes transactivated by this transcriptional factor (107). To summarize, both T1DM and T2DM are featured by autophagic inhibition but at chronic stages of the disease.

AMP-activated protein kinase is an important sensor of cell energy status (101). AMPK activation by an increase in the AMP/ATP ratio stimulates autophagic flux (101). AMPK activity is inhibited in STZ + HFD-induced T2DM mice. Furthermore, in HFD-fed mice, AMPK activity is slightly inhibited and the decrease in AMPK activity is related to a decrease in autophagic flux, measured by the autophagic marker LC3 II (108, 109). Treatment with sulforaphane, a naturally occurring isothiocyanate in plants, positively regulates the expression of Nrf2 and its downstream genes. This compound prevents diabetes-induced AMPK inhibition associated with an increase in LC3 II (108). On the other hand, in STZ-induced T1DM mice model, the inhibition of autophagic flux by AMPK 6 months after the onset of diabetes is described (106). This finding is also observed in H9C2 cells when autophagic flux is evaluated using a mCherry-Gfp-Lc3 construct (106). All these findings support the idea that autophagy is inhibited in the chronic stages of both T1DM and T2DM (101, 110, 111). Because there is altered energy metabolism and dysfunctional insulin signaling, the accumulation of metabolic substrates produced by hyperlipidemia and hyperglycemia in the chronic stages of TD1M and T2DM can lead to the inhibition of AMPK activity with the consequent inhibition of autophagic flow. However, the use of LC3 II as the only marker of autophagy should be done cautiously (104). In hyperlipidemia-dependent T2DM mice models, such as those generated using HFD, LC3 II is also located on the surface of lipid droplets (112). Therefore, AMPK and LC3 II changes could be related to the selective degradation of lipid droplets in the cytoplasm of cardiac cells rather than associated to autophagosome formation.

Several cardioprotective molecules regulating the autophagic flux in DCM have been described. Lin-28 homolog A (Lin28a) is decreased in high glucose conditions and inhibits the mammalian sterile 20-like kinase 1 (Mst1) (113). Lin28a overexpression activates autophagy in cardiomyocytes (113). Interestingly, the Lin28a-dependent protective effect against high glucose conditions was abolished when autophagy was inhibited by 3-metil-adenine. This data suggest that autophagy is involved in *in vitro* cardio protection (113). miR34a is upregulated in the myocardial tissue of diabetic patients and mice, leading to DCM (114). Flavanol (dihydromyricetin) and astragaloside-IV (triterpenoid saponin) downregulate miR34a and ameliorate DCM in STZ-treated rats in an autophagy-dependent mechanism (114, 115). Although, in these papers, the exact mechanism by which miR34a affected the autophagic flux was not disclosed, Pang et al. described in other models that miR34a targeted autophagy-related 9 A (ATG9A) (116). miR207 that targets LAMP2 is also upregulated and inhibits the autophagic flux in DCM (117). However, the inhibition of miR207 or the overexpression of LAMP2 for reverting the DCM phenotype is still not examined *in vivo* (117).

Cardiokines, which are defined as peptides released from the heart (118), are also an emerging topic in DCM. IL-33, a known cardiokine downregulated in the diabetic heart, prevents the onset of diastolic dysfunction in db/db mice through autophagy and endoplasmic reticulum stress-dependent mechanisms (119). Moreover, fibroblast growth factor 21 (FGF21), another cardiokine, prevents the systolic dysfunction associated to DCM in STZ-treated animals (120, 121).

**Figure 3:**
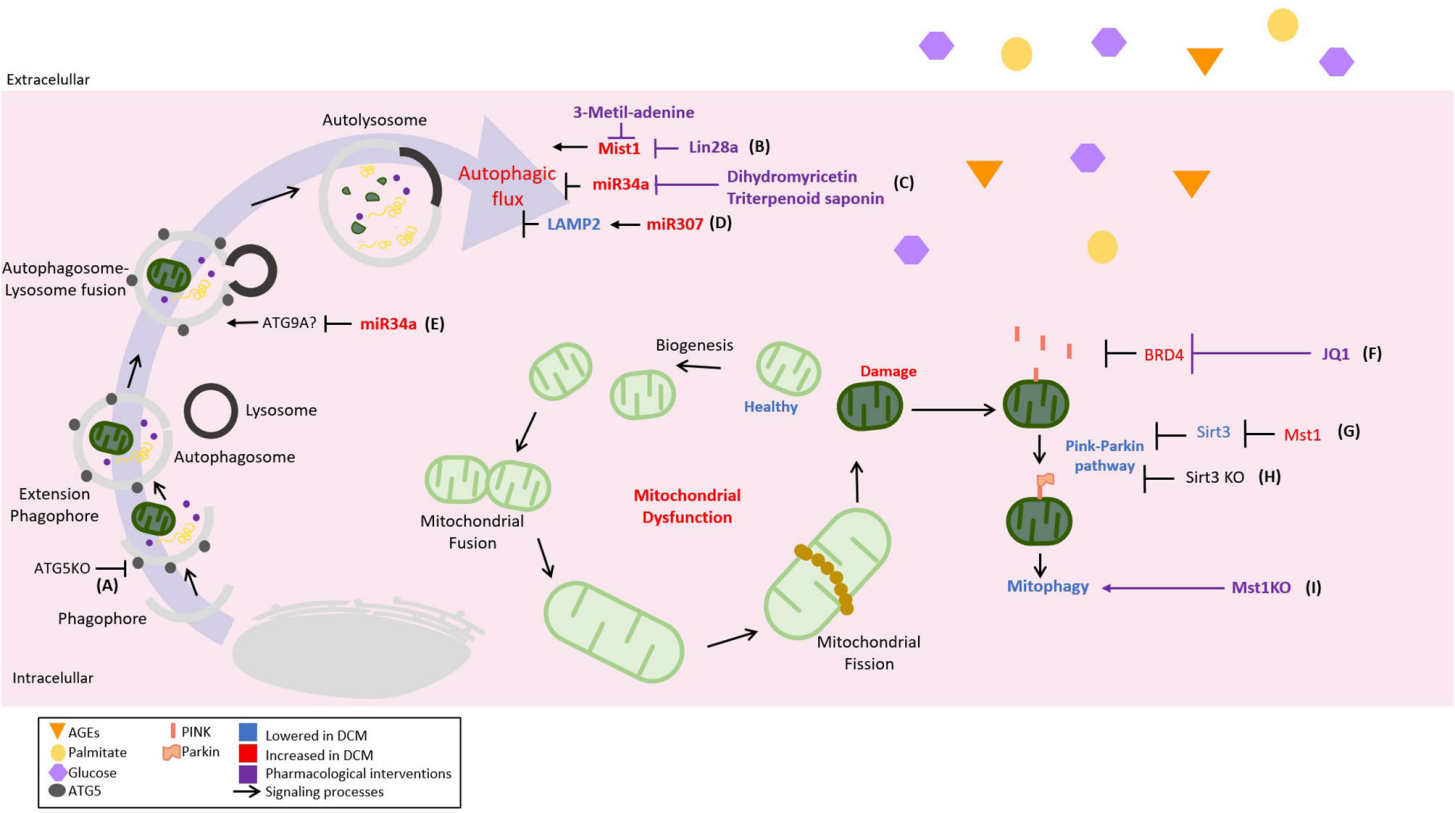
Novel regulations of cellular catabolic pathways in DCM and its therapeutical strategies. The autophagic inhibition plays a critical role in the development and progression of DCM; therefore, different pathological mechanisms and their pharmacological strategies have been described. It can be illustrated as follows: **(A)** acceleration of cardiac dysfunction progression in type 1 diabetes mellitus (T1DM) with autophagy-related gene 5 (ATG5) KO, **(B)** the promotion of cardiac dysfunction by mammalian sterile 20-like kinase 1- (Mst1-) inhibited autophagic flow. Lin-28 homolog A (Lin28a) can inhibit Mst1 activating autophagy, **(C)** the inhibition of autophagic flow by the upregulation of miR-34a in the diabetic heart. As a pharmacological strategy, dihydromethicetin decreases the expression of miR-34a, restoring the impaired autophagy, **(D)** diabetic heart upregulates miR-207, which inhibits the autophagic flow in an LAPM2-dependent mechanism, and **(E)** miR-34a could inhibit the autophagic flux by targeting autophagy-related 9 A (ATG9A). Mitophagy, the mechanism by which damaged or defective mitochondria are eliminated, is strongly inhibited in DCM, being considered a pathological feature. For this reason, mechanisms and therapeutic strategies aiming at mitophagy recovery have also been included and represented as follows: **(F)** the inhibition of mitophagy by bromodomain-containing protein 4 (BRD4) that is restored by JQ1 in a PTEN-induced kinase 1- (PINK1-) dependent mechanism, **(G)** Mst1 decreases mitophagy using a PARKIN-dependent mechanism by inhibiting sirtuin 3 (SIRT3) expression; therefore, **(H)** SIRT3 KO reduces mitophagy also in a Parkin-dependent manner, and **(I)** Mst1 KO to induce mitophagy.

Despite the defective autophagic flux in DCM and the success achieved for some interventions aiming for restoration (Figure 3), it is still a matter of discussion, which are the main molecular targets downregulated among the many proteins participating in the autophagic process. Knowledge about the precise molecular targets could be an invaluable tool for the future development of therapies.

Moreover, according to the preclinical data, an increase of autophagy in human right atrial samples derived from patients with T2DM is also described. Electron microscopy shows a larger number of autophagosomes associated with increased levels of LC3B-2 and Beclin-1 (122). Conversely, a recent report using atrial samples derived from patients with T2DM who underwent cardiopulmonary bypass showed that autophagy was suppressed when compared with the samples from nondiabetic patients, measured using Beclin-1 and ATG4 levels (123). On the other hand, the samples from diabetic HF patients submitted to the left ventricle-assisted device (LVAD) have shown that several autophagic markers such as LC3B, ATG3, and Beclin-1 were upregulated and associated with a decrease in the levels of miR-133a when compared with non-diabetic HF patients (124). Therefore, most of the preclinical and clinical data show that autophagy plays a role in the pathogenesis of cardiac complications of diabetes. However, the controversy of whether it is upregulated or downregulated still remains to be elucidated. Furthermore, the available data have led us to think that the role of autophagy is far more complex than merely a regulation of activation level. It seems to be related to the physio-pathological stage.

### Impaired Mitophagy as a Feature of DCM

Mitophagy is a specialized catabolic process aiming to eliminate defective or damaged mitochondria through autophagy. This process is triggered by a decrease in the mitochondrial membrane potential, which stabilizes in the OMM the serine-threonine kinase PTEN-induced kinase 1 (PINK1), which in turn recruits the cytosolic protein E3-ligase Parkin (102). The PINK1/Parkin pathway functions as a molecular tag for the removal of damaged mitochondria. Mitophagy is essential in the maintenance of healthy cardiomyocytes, and defective mitophagy is a feature of several cardiac diseases, including DCM (94).

PTEN-induced kinase 1-Parkin-mediated mitophagy was suppressed in DCM induced by HFD (125, 126). In diabetic mouse hearts, the upregulation of bromodomain-containing protein 4 (BRD4), a member of the BET family of epigenetic regulators, inhibits PINK1/Parkin-mediated mitophagy, triggering the accumulation of damaged mitochondria with the subsequent impairment of cardiac structure and function (125). The inhibition of BRD4 with JQ1 restores mitochondrial function, cardiac structure, and the systolic and diastolic function through a PINK1-dependent mechanism (125). In HFD mice, the deletion of Parkin partially inhibits mitophagy and triggers a worsening of myocardial lipid accumulation and diastolic dysfunction (126). Moreover, the restoration of Sirt3-Foxo3A-Parkin signaling upregulates mitophagy and protects the development of DCM in STZ mice (127). On the other hand, Mst1 can induce DCM in a Parkin-dependent mechanism. Mst1 inhibits Sirt3 expression leading to the downregulation of Parkin, cardiomyocyte mitophagy inhibition, and DCM development (128). Melatonin administration inhibits Mst1 and activates Parkin pathway. This treatment rescues the impairment of cardiac mitophagy and alleviates systolic disfunction associated with DCM (129). The role of Mst1 in the development of DCM is further corroborated in the Mst1 knockout mice. The deletion of Mst1 gene reduces mitochondrial fission and alleviates left ventricular remodeling and systolic dysfunction in diabetic mice (130). Most of the previous studies are conducted using STZ mice models. However, in a db/db mice model, Mst1 knockdown also alleviates cardiac lipotoxicity and inhibits the development of DCM (131). These data suggest that Mst1/Parkin is a common and general mechanism for developing DCM in both T1DM and T2DM. Furthermore, despite the interesting data from animal models (Figure 3), there is a lack of knowledge about the clinical relevance of mitophagy stimulation as a therapy in DCM.

## Discussion and Future Perspectives

Diabetic cardiomyopathy is characterized by several cellular and molecular features. The low-grade inflammation appears to be central as several experimental anti-inflammatory approaches, particularly involving the TLR4/MD2 pathway, prevent the development of DCM (19). Moreover, systemic interference of TLR4 expression using siRNA also prevents the development of DCM in STZ-treated mice (21). Despite these successful interventions, there are only a few articles linking TLR signaling with the pathogenesis of cardiac diseases. On the other hand, although most of the available data connect the NRLP3 inflammasome with the induction of DCM, probably other inflammasome complexes could also be involved in the DCM physiopathology, i.e., AIM2 inflammasome (35). Therefore, more research is required to clarify the role of all inflammasome complexes and their utility as a target for novel therapies for DCM. The increasing body of evidence, showing the strategies that successfully attenuated DCM by targeting inflammation (Figure 1), has converted this topic into a promising source for pharmacological interventions.

Despite the many functions described for miR presented earlier, other protective miR approaches have also been described in T1DM genetic models using the Akita mice. In these papers, the overexpression of miR-133a improves a systolic function and prevents structural remodeling, suggesting that miR-133a has protective actions in DCM (132, 133). However, it is also important to go deeper into the molecular mechanisms of the protective effects of miR-133a and is necessary to evaluate whether the intervention can successfully attenuate the DCM phenotype in other animal models.

The myocardial reprogramming on mitochondrial substrate utilization from glucose oxidation toward fatty acid oxidation has always been considered as a pathophysiological feature that could be responsible for the development of DCM. However, because of the latest information, this metabolic change is now seen as an adaptation to limit glucotoxicity in the heart (66). Currently, another change in myocardial substrate utilization, the ketone bodies, is becoming the main interest. Clinical trials using SGLT2i suggest that the increase of ketone body utilization in the diabetic heart could be protective. It remains to be elucidated if the modulation of OGA modifies the ketone body metabolism by preventing the OGA of key enzymes in the pathway. Although the pharmacological interest has clearly focused on the modulation of ketone metabolism, this evidence suggests that metabolic alterations have a causal role in the development of the DCM phenotype.

Regarding mitochondrial dynamics, OPA1 and Mfn2 are possible targets to prevent DCM development (97, 99). As several interventions modulating mitochondrial physiology achieved the goal of modifying the fate of the DCM phenotype, they could be considered as pharmacological strategies (Figure 2). However, pharmacological interventions to modulate mitochondrial dynamics suitable to perform clinical trials still need to be developed.

In DCM, the catabolic pathways such as autophagy or mitophagy are strongly suppressed (106). The mentioned suppression is probably propitiating the aberrant signaling of known cellular mediators like the cited example of Nrf2. That led us to think that there will be more examples of aberrant signaling implicated in DCM, once the inhibition of recycling pathways has taken place. For the mentioned reasons, more experimental interventions aiming to normalize autophagic flux are required in DCM. In the case of mitophagy, the evidence is strongly pointing to decreased PINK1/Parkin pathway (Figure 3), and future interventions aiming to recover its activity of improving mitochondrial quality control must be taken in consideration to look for a clinical application.

Cardiac delivery is a current challenge of DCM pharmacology. Several approaches are currently used to ensure the cardiac delivery of therapeutic agents, thus overcoming possible side effects produced by an untargeted distribution (134). However, the application of cardiac selective delivery to DCM is still not a widely used strategy. Conversely, accomplishing the criteria of attenuating or preventing the DCM phenotype is achieved in several examples. Nanotechnology has been used to selectively deliver the therapeutic molecules. For instance, liposomes loaded with a non-mitogenic acid fibroblast growth factor and associated to a microbubble are designed to be precisely destroyed with the targeted ultrasound. This approach led to an improved systolic function and milder structural remodeling in STZ-treated rats (135). Effective cardiac gene delivery using the natural tropism of adeno-associated virus serotypes 9 and 6 toward the DCM myocardium are described (89, 136, 137). The clinical safety of adeno-associated vectors has been tested in many clinical trials, and there are drugs using this technology on the market (138). For this reason, gene therapy using this technology is a promising tool to achieve the goal of cardiac specific delivery in DCM.

Several antidiabetic agents have been demonstrated to be cardioprotective in a clinical setup, including several ISGLT2 inhibitors, liraglutide, semaglutide, and metformin. However, the reported benefits of these molecules are in HF patients and there is scarce information available specifically for DCM. One of the available studies shows that dapagliflozin produces a regression in the increased left ventricular mass of patients with T2DM (139). A similar finding is reported for gliclazide when compared to other antidiabetic agents such as glyburide, voglibose, metformin, pioglitazone, rosiglitazone, and sitagliptin. A small clinical trial with liraglutide also shows the improvement of a diastolic function but is still not clear if this improvement delays the onset of symptomatic DCM (140, 141). Conversely, other drugs like exantenide and sitagliptin do not improve the systolic or diastolic dysfunction in patients with T2DM (142, 143). However, sitagliptin is used to prevent the exacerbation of DCM in T2DM when it is used together with other antidiabetic treatments (144). Finally, it is important to take a second look at the already approved therapies in the search for new DCM drugs because not only some of them can be useful but also the study of the mechanisms can lead to the discovery of new molecules with better therapeutical properties.

In total, the presented data led us to propose that for the case of DCM having a bunch of cellular and molecular alterations taking place at the same time in the myocardium, it is convenient to explore the health of broad cellular processes like the ones mentioned earlier because many of the cardinal features of the diabetic heart may be consequences of alterations of broad cellular pathways. Thus, the approaches capable of alleviating these alterations give us a better chance to achieve the goal of a new treatment.

## Author Contributions

FM-C, CH-F, CL-C, MT, XC, and AG-M wrote the manuscript. LG, MC, PC, and SL designed, supervised, revised the manuscript, and approved the final version. All authors contributed to the article and approved the submitted version.

## Funding

This work was supported by grants from the Agencia Nacional de Investigacion y Desarrollo (ANID), Chile: FONDECYT (1180157 to MC, 3190546 to CL-C, 120049 to SL, and 1181097 to PC), FONDAP (15130011 to MC and SL), and ANID fellowships (21181428 to FM-C, 21180537 to MT, and 21201710 to XC).

## Conflict of Interest

The authors declare that the research was conducted in the absence of any commercial or financial relationships that could be construed as a potential conflict of interest.

## Publisher's Note

All claims expressed in this article are solely those of the authors and do not necessarily represent those of their affiliated organizations, or those of the publisher, the editors and the reviewers. Any product that may be evaluated in this article, or claim that may be made by its manufacturer, is not guaranteed or endorsed by the publisher.
